# Amniotic fluid embolism Number 11 – 2026

**DOI:** 10.61622/rbgo/2026FPS11

**Published:** 2026-08-13

**Authors:** Álvaro Luiz Lage Alves, Alexandre Massao Nozaki, Lucas Barbosa da Silva

**Affiliations:** 1 Hospital das Clínicas Universidade Federal de Minas Gerais Belo Horizonte MG Brasil Hospital das Clínicas, Universidade Federal de Minas Gerais, Belo Horizonte, MG, Brasil.; 2 Hospital das Clínicas Faculdade de Medicina Universidade de São Paulo São Paulo SP Brasil Hospital das Clínicas, Faculdade de Medicina, Universidade de São Paulo, São Paulo, SP, Brasil.; 3 Hospital das Clínicas Universidade Federal de Minas Gerais Belo Horizonte MG Brasil Hospital das Clínicas, Universidade Federal de Minas Gerais, Belo Horizonte, MG, Brasil.

## Abstract

•Amniotic fluid embolism is a rare condition with high maternal mortality. It typically occurs during labor or delivery, and the pathogenesis has not yet been fully elucidated.

•Survival of parturients with amniotic fluid embolism requires early recognition, rapid initiation of treatment, and access to skilled care based on the management of critically ill obstetric patients.

•The classic clinical presentation of amniotic fluid embolism involves sudden hypoxia and hypotension or cardiopulmonary arrest, accompanied or immediately followed by non-cardiogenic pulmonary edema and hemorrhage secondary to disseminated intravascular coagulation.

•In cases of amniotic fluid embolism occurring during labor, bleeding typically originates at invasive interventions sites (intravascular lines and urinary catheterization). Following vaginal or cesarean delivery, bleeding arises primarily from the uterus, birth canal lacerations, and incision sites (abdominal wall and episiotomies).

•Key laboratory abnormalities observed in amniotic fluid embolism include decreased fibrinogen levels, thrombocytopenia, and elevated D-dimer levels. The latter has limited diagnostic value, as levels are typically elevated during pregnancy and the postpartum period.

•Arterial blood gas analysis in amniotic fluid embolism indicates the presence of hypoxemia and metabolic acidosis. Although chest radiography is initially unremarkable, if pulmonary edema and respiratory distress syndrome are present, dense bilateral pulmonary infiltrates may be observed.

•The identification of amniotic fluid debris in pulmonary artery catheters or histopathological samples of lung tissue should not be considered diagnostic of amniotic fluid embolism.

•The primary goals in managing amniotic fluid embolism are cardiopulmonary resuscitation, hemorrhage control, reversal of coagulopathy, and when indicated, fetal delivery.

•In cases of amniotic fluid embolism accompanied by coagulopathy, massive transfusion is a necessary measure for effective, definitive treatment. The infusion of uterotonics, non-surgical invasive interventions (intrauterine balloons and intrauterine suction systems), and surgical procedures (vascular ligation, uterine compression sutures, and hysterectomy) may be used as adjuncts to control hemorrhage but are not considered definitive treatments.

•The main differential diagnoses for amniotic fluid embolism include complications of neuraxial anesthesia; hemorrhage due to uterine atony, retained products of conception, and/or birth canal trauma; placental abruption; pulmonary thromboembolism; air embolism; acute myocardial infarction; and anaphylactic, cardiogenic, or septic shock.

## Recommendations

- Sudden cardiopulmonary arrest or arterial hypotension associated with acute respiratory failure, disseminated intravascular coagulation, clinical onset during labor or within 30 minutes of placental delivery, and the absence of fever during labor are the four non-exclusive criteria that can be used to diagnose amniotic fluid embolism.
- The management of amniotic fluid embolism requires a rapid multidisciplinary approach and should ideally involve obstetricians, neonatologists, anesthesiologists, and intensivists, as well as nursing staff.
- In cases of amniotic fluid embolism, basic and advanced cardiovascular life support procedures must be initiated immediately to reverse shock and cardiopulmonary arrest. This requires effective chest compressions, rapid airway management involving ventilation and endotracheal intubation, and the establishment of supradiaphragmatic venous access for fluid replacement and pharmacological treatment of cardiac arrhythmias.
- Cardiopulmonary resuscitation for patients with amniotic fluid embolism must follow advanced cardiovascular life support guidelines. Chest compressions should be performed with the pregnant woman in the supine position. The compression rate should be between 100 and 120 compressions per minute, and the chest should be compressed to a depth of 5 to 6 cm.
- In amniotic fluid embolism, manual uterine displacement during cardiopulmonary resuscitation is frequently recommended, as most cases occur during the later stages of pregnancy, with the uterine fundus located at or above the level of the umbilicus.
- Hemodynamic support for amniotic fluid embolism involves the administration of fluids and vasopressors. Initial resuscitation should be carried out via rapid intravenous fluid bolus (500 mL). In patients showing evidence of hemorrhage, blood is preferable to crystalloids. Vasopressor therapy is appropriate for patients who remain hypotensive following adequate initial resuscitation, and norepinephrine is the first-line agent.
- Respiratory failure associated with amniotic fluid embolism should be treated with supplemental oxygen and, in most cases, endotracheal intubation and mechanical ventilation. Bag-valve-mask ventilation with 100% oxygen should be used while preparations for endotracheal intubation are made. Supraglottic devices serve as effective alternatives in the event of intubation failure. Before intubation, two ventilations are recommended for every 30 chest compressions. After intubation, ventilations should be continuous, at a rate of 8 to 10 per minute.
- In cases of amniotic fluid embolism progressing to severe hemorrhage and/or disseminated intravascular coagulation, massive blood transfusion is essential. Crystalloids should be infused until blood components become available, with a maximum recommended infusion volume of 1,500 mL.
- Immediate delivery may be necessary for amniotic fluid embolism occurring after 22–23 weeks of gestation. The most common factors requiring urgent delivery are non-reassuring fetal status and rapid, progressive deterioration of the mother’s clinical condition.
- Following cardiopulmonary resuscitation, hemorrhage control, reversal of coagulopathy, and delivery, ongoing care for amniotic fluid embolism must take place in an intensive care unit. Clinical support should focus on maintaining a secure airway, hemodynamic stability and adequate oxygenation, as well as monitoring for and preventing hemorrhage.

## Background

Amniotic fluid embolism (AFE) is a rare condition associated with high maternal mortality. It typically occurs during labor or delivery. Its pathogenesis is not yet fully understood but appears to involve a ‘cytokine storm’ triggered in response to exposure to an unknown antigen, likely present in the amniotic fluid.^(1,2)^

Survival for parturient women affected by AFE requires early recognition, rapid implementation of potentially life-saving therapies, and access to skilled care based on the competent management of critically ill obstetric patients.^(1,2)^

### What is the incidence of amniotic fluid embolism?

The incidence of AFE is prone to overestimation. Since its recognition depends on diagnostic criteria and the exclusion of other etiologies presenting with similar clinical findings, it is always possible that other conditions progressing to cardiopulmonary arrest (CPA) and disseminated intravascular coagulation (DIC) are recorded as AFE, particularly when heterogeneous criteria are used to establish the diagnosis.^(3)^

A review of reports from Australia, Canada, the United Kingdom, and the United States found an AFE incidence ranging from 1.9 to 6.1 cases per 100,000 deliveries.^(4)^

### What are the key aspects of the pathogenesis of amniotic fluid embolism?

The pathogenesis of AFE has not yet been fully elucidated. The leading pathogenic hypothesis posits a biphasic model. The first phase is characterized by the entry of amniotic fluid containing fetal cells and other antigenic products into the maternal systemic circulation through a disruption at the maternal-fetal interface. In the second phase, there is abnormal activation of humoral and immunological processes, involving the release of vasoactive and procoagulant substances, similar to the mechanisms seen in systemic inflammatory response syndrome and anaphylactic shock. For this reason, AFE is also referred to as anaphylactoid syndrome of pregnancy.^(2)^

The clinical presentation progresses to acute pulmonary hypertension and right ventricular failure due to pulmonary artery vasospasm (acute cor pulmonale). Subsequently, right ventricular insufficiency leads to left ventricular failure, systemic hypotension, and CPA. Left ventricular insufficiency may also result from the direct effect of hypoxia, the release of maternal inflammatory mediators, or a direct depressant effect of amniotic fluid on the myocardium.^(2)^

Acute pulmonary hypertension results in hypoperfusion, cardiogenic pulmonary edema, and respiratory failure. Some patients develop non-cardiogenic pulmonary edema. This progression is explained by damage to the endothelial-alveolar membrane and capillary leak syndrome, corroborated by the presence of high protein concentrations in the edema fluid and amniotic fluid debris in the pulmonary secretions and alveolar spaces of these patients.^(2)^

Abnormal activation of humoral and immunological processes, involving the release of vasoactive and procoagulant substances, results in DIC. The pathogenic process appears to involve the activation of factor VII and platelets, as well as the release of inflammatory mediators. Together, these activate the coagulation cascade, leading to DIC, ischemic dysfunction, and multiple organ failure. In turn, hemorrhage resulting from DIC exacerbates hemodynamic instability.^(1,2)^

Just like the detection of fetal cells and other amniotic fluid debris in pulmonary autopsy samples, the changes observed in inflammatory markers, specifically reduced complement levels (C3, C4, and C1 esterase inhibitor), elevated serum tryptase levels, and increased pulmonary mast cell activity, lack diagnostic value. This limitation arises because these markers were benchmarked against healthy pregnant controls rather than critically ill patients with other obstetric or systemic emergencies.^(2,5)^

### What are the main clinical aspects of amniotic fluid embolism?

Most cases of AFE occur during labor and delivery (70%) or within 30 minutes of birth.^(2)^ Rare cases of AFE have been reported complicating first- and second-trimester abortions (medical or surgical), amniocentesis, and abdominal or uterine trauma.^(6-8)^

In most cases (90%), the clinical presentation of AFE is abrupt, rapidly progressive, and extremely severe. The classic clinical picture involves sudden hypoxia and hypotension or CPA, accompanied or immediately followed by non-cardiogenic pulmonary edema and hemorrhage secondary to DIC. In cases of AFE occurring during cesarean deliveries, the clinical course may be slower or even attenuated due to initial anesthetic interventions implemented to correct early vital sign changes presumed to be related to the anesthesia.^(2,9)^

The main clinical manifestations include a prodromal phase (aura), respiratory failure or CPA, hemorrhage, and, more rarely, tonic-clonic seizures and/or stroke. The prodromal phase presents as a sense of impending doom, chills, nausea, vomiting, agitation, anxiety, or altered mental status, manifesting immediately before CPA. Hypoxemic respiratory failure and hypotension due to cardiogenic shock, which frequently culminate in cardiac arrest, manifest as oxygen desaturation, dyspnea, tachypnea, cyanosis, crackles, and, less commonly, wheezing. Cardiac arrest occurs due to pulseless ventricular tachycardia or ventricular fibrillation, but it can also be caused by asystole or bradyarrhythmia. Patients who survive CPA typically develop non-cardiogenic pulmonary edema, with worsening dyspnea, tachypnea, and crackles. Hemorrhage due to DIC occurs in 80% of patients with AFE, typically shortly after the onset of cardiopulmonary compromise. In cases of DIC occurring during labor, bleeding commonly originates from sites of invasive interventions (intravascular catheters, bladder catheterization). Following vaginal or cesarean delivery, bleeding arises primarily from the uterus, birth canal lacerations, and incision sites (abdominal wall and episiotomies).^(1,2,9)^

Rare cases of AFE with overt DIC in the absence of prior cardiopulmonary compromise have been reported.^(10)^ Tonic-clonic seizures and/or stroke may occur in AFE, though these are rare manifestations and uncommon complications. Milder, atypical cases of AFE have also been described, characterized by the incomplete presentation of classic signs and symptoms, a shortened clinical course, and a better prognosis.^(2,11)^

### What are the key laboratory and imaging findings in amniotic fluid embolism?

In AFE, the main laboratory abnormalities are related to DIC, such as elevated D-dimer, hypofibrinogenemia (<200 mg/dL), and thrombocytopenia, which typically appear within 30 minutes of the onset of cardiopulmonary compromise. Because D-dimer levels are commonly elevated during pregnancy and the postpartum period, the diagnostic value of this marker is limited.^(2,9)^ When available, thromboelastography can assist in identifying coagulopathy in AFE patients who do not show overt signs of bleeding.^(12)^

Other laboratory findings in peripheral blood samples include a drop in hemoglobin and hematocrit levels secondary to hemorrhage, as well as leukocytosis. The latter is a nonspecific finding, given that it also occurs in healthy women during labor and the postpartum period.^(2,9)^

Arterial blood gas analysis typically indicates hypoxemia and metabolic acidosis resulting from prolonged hypotension and/or CPA. Chest radiography may appear normal during the initial stages of AFE. If pulmonary edema and respiratory distress syndrome are present, dense bilateral pulmonary infiltrates may be observed. Transthoracic or transesophageal echocardiography reveals elevated pulmonary pressures during the initial phase (lasting 15 to 30 minutes), characterized by a severely hypokinetic and dilated right ventricle that causes the interventricular septum to shift toward the left ventricle. This is followed by a reduction in left ventricular ejection fraction. Prior to CPA, the electrocardiogram usually shows sinus tachycardia. In cases of CPA, tracings reveal the rhythms mentioned above; pulseless ventricular tachycardia and ventricular fibrillation are the most common.^(1,2,9)^

In cases of AFE occurring during labor, the impact on fetal oxygenation is acute and severe. Maternal cardiopulmonary compromise and catecholamine release reduce uteroplacental perfusion and increase uterine contractility and tone, leading to fetal hypoxemia and acidosis. These effects can be observed on cardiotocography as a lack of fetal heart rate variability, late decelerations, or terminal bradycardia. Severe fetal hypoxia may precede maternal cardiopulmonary collapse.^(2,9)^

### How is the clinical diagnosis of amniotic fluid embolism made, and what are the diagnostic criteria?

The clinical diagnosis of AFE is based on the presence of clinical findings combined with the exclusion of other etiologies that present with similar features. Classic clinical presentations involve patients in labor or in the immediate postpartum period who experience sudden CPA, respiratory failure, and/or seizures, particularly those who subsequently develop DIC. However, not all of these manifestations are necessarily present in patients with AFE. Many diagnoses are made retrospectively, only after data analysis and the review of autopsy findings. Atypical cases may present solely with respiratory failure and hypotension. Rarely, DIC may be the initial clinical manifestation or, conversely, may be absent.^(2,9)^

The identification of amniotic fluid debris (squamous cells, trophoblastic cells, mucin, and lanugo) in blood drawn from the tips of pulmonary artery catheters or in histopathological samples of lung tissue should not be considered diagnostic, as these findings can be present in the maternal circulation and lungs of patients without AFE.^(13)^ Additionally, many patients with the classic clinical presentation do not show histological evidence of these amniotic fluid products at autopsy.^(14)^

The presence of four non-mutually exclusive diagnostic criteria has been proposed for the definition of AFE.^(15)^ The criteria, all of which must be present, include:

- Sudden CPA or arterial hypotension (systolic blood pressure < 90 mmHg) associated with acute respiratory insufficiency (dyspnea, cyanosis, and peripheral oxygen saturation < 90%).
- Disseminated intravascular coagulation documented via a scoring system using: (a) thrombocytopenia (>100,000/mL = 0 points; between 50,000 and 100,000/mL = 1 point; < 50,000/mL = 2 points); (b) prothrombin time prolongation or international normalized ratio (INR) (<25% = 0 points; increase between 25% and 50% = 1 point; increase > 50% = 2 points); and (c) fibrinogen levels (>200 mg/dL = 0 points; < 200 mg/dL = 1 point). A score of ≥ 3 points is consistent with overt DIC.^(16)^
- Clinical onset during labor or within 30 minutes of placental delivery.
- Absence of fever (≥38 °C) during labor.

The presence of all four criteria rules out patients without AFE, meaning it has a strong negative predictive value, though it may fail to diagnose atypical cases.^(17)^

### What should initial management of amniotic fluid embolism entail?

Managing AFE requires a rapid, multidisciplinary approach. Ideally, care should involve obstetricians, neonatologists, anesthesiologists, intensivists, and nursing professionals. The primary goals are cardiopulmonary resuscitation (CPR), hemorrhage control, reversal of coagulopathy, and when indicated, fetal delivery.^(18)^

Initial management must focus on high-quality CPR, featuring rapid (100–120 compressions per minute) and vigorous (5–6 cm deep) chest compressions.^(9)^ Administration of tranexamic acid (1 g intravenously over 10 minutes) and activation of a massive transfusion protocol must be immediate.^(19,20)^ Fetuses at a gestational age of ≥22–24 weeks should be delivered to optimize maternal resuscitation (via aortocaval decompression). If the fetus is alive, delivery also facilitates neonatal resuscitation and survival. Ruling out other etiologies supports a presumptive diagnosis of AFE and should also be part of the initial approach.^(9)^

### What should cardiopulmonary resuscitation entail in cases of amniotic fluid embolism?

Basic and Advanced Cardiovascular Life Support (BLS and ACLS) procedures must be initiated immediately to reverse shock and CPA, ensuring tissue perfusion for both mother and fetus. To this end, efficient chest compressions, rapid airway management (including ventilation and endotracheal intubation), and the establishment of supradiaphragmatic venous access for fluid resuscitation and pharmacological treatment of cardiac arrhythmias are required.^(18)^

Since AFE in pregnancy typically occurs during the later stages of gestation, specific details regarding CPR in this context are essential. Uteri with a fundal height at or above the level of the umbilicus must be manually displaced to the left. This maneuver aims to reduce aortocaval compression, optimize venous return (preload), and maintain the upper torso in a supine position, thereby preserving the efficacy of chest compressions and adequate stroke volume. To achieve this, a one- or two-handed manual uterine displacement technique should be implemented, with one or both hands positioned on the right lateral border of the uterus to shift it laterally to the left, approximately 3 to 4 cm away from the midline (Figure 1). In addition to optimizing chest compressions, manual uterine displacement facilitates airway management and the establishment of venous access.^(20)^

**Figure 1:**
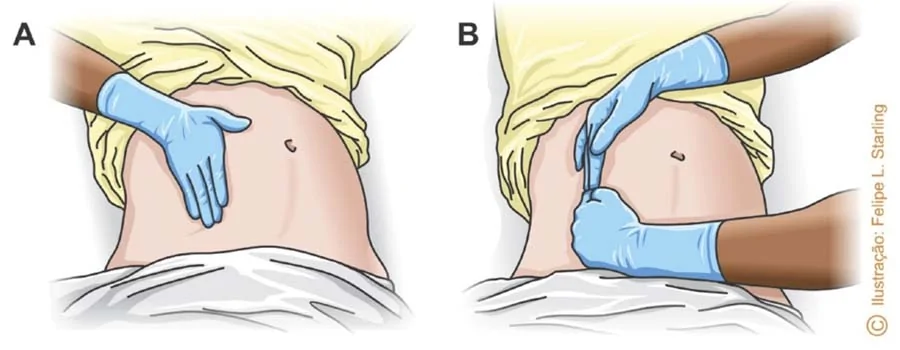
Technique for manual uterine displacement using one hand (A) or two hands (B).

Source: Illustration by Felipe Lage Starling (used with permission).

High-quality chest compressions are essential and must be performed with the pregnant woman in the supine position. The use of a firm surface beneath the chest is indicated but should not delay the initiation of resuscitation. The compression rate should be between 100 and 120 compressions per minute, with a depth of 5 to 6 cm per downstroke. Hands should be positioned in the center of the chest, over the lower half of the sternum; no adjustments are required for pregnancy. After each compression, full chest recoil must be allowed, avoiding continuous leaning on the patient’s torso. Chest compressions should be uninterrupted, except during defibrillation and pulse checks (in the absence of continuous end-tidal CO_2_ monitoring), when indicated.^(20,21)^

During the second half of pregnancy, venous access should be established above the diaphragm, as medications administered via the femoral vein may not reach the maternal heart until the fetus is delivered. Obtaining dual access via the antecubital veins using 14-gauge catheters (e.g., Jelco or Abocath) is as effective for volume replacement as central access, although it lacks hemodynamic monitoring capabilities. Therefore, time should not be wasted, nor resuscitation interrupted, to secure central venous access, as this, like monitoring catheters, can be established after the patient has been stabilized.^(20)^

Hemodynamic support involves the administration of fluids and vasopressors. Initial resuscitation should be carried out with rapid intravenous fluid infusion to restore tissue perfusion. Crystalloids (lactated Ringer’s and normal saline) are recommended. They should be administered in 500 mL boluses with frequent assessment of the patient’s response through vital sign monitoring. Colloids should be avoided. In patients with evidence of hemorrhage, blood products are preferable to crystalloids, although crystalloid infusion is indicated until blood transfusion becomes feasible. Fluid administration should be discontinued once intravascular volume has been restored or if pulmonary edema develops.^(20)^ Vasopressor therapy is appropriate for patients who remain hypotensive following adequate initial resuscitation. Norepinephrine is the first-line agent. However, the addition of dobutamine may be beneficial in patients with concomitant cardiogenic shock.^(22)^

Patients with persistent cardiogenic shock due to right ventricular failure (acute cor pulmonale) require inotropes. Dobutamine and milrinone are the preferred agents for improving right ventricular output. Sildenafil (oral or via orogastric tube), prostacyclin (epoprostenol; intravenous or inhaled), and nitric oxide (inhaled) are recommended to reduce pulmonary vascular resistance, thereby contributing to the reversal of cardiocirculatory collapse. Chart 1 lists the main drugs indicated for CPR due to AFE.^(1)^

**Chart 1:** Main drugs indicated for cardiopulmonary resuscitation due to amniotic fluid embolism

Drug	Dosage
Norepinephrine (vasopressor)	0.05–3.3 µg/kg/min
Dobutamine (inotropic)	2.5–5.0 µg/kg/min
Milrinone (inotropic)	0.25–0.75 µg/kg/min
Sildenafil (pulmonary vasodilator)	20 mg orally every 8 hours
Inhaled nitric oxide (pulmonary vasodilator)	5–40 parts per million (ppm)
Inhaled prostacyclin (epoprostenol) (pulmonary vasodilator)	10–50 ng/kg/min
Intravenous prostacyclin (epoprostenol) (pulmonary vasodilator)	Start with 1 to 2 ng/kg/min and titrate to desired effect

Source: Adapted from Pacheco et al. (2020).^(1)^

Respiratory failure must be treated with supplemental oxygen and, in most cases, endotracheal intubation and mechanical ventilation. In AFE presenting with CPA, endotracheal intubation should be performed early and requires a skilled professional. In advanced pregnancy, both intubation and bag-valve-mask ventilation are more difficult due to physiological edema, airway narrowing, and reduced chest wall compliance. Bag-valve-mask ventilation with 100% O_2_ (8 to 10 breaths/minute, using a two-handed technique, >15 liters per minute) combined with airway suctioning is the fastest, albeit less efficient, strategy. It should be used while preparing for endotracheal intubation to prevent desaturation. Prolonged intubation attempts must be avoided; a maximum of two attempts is recommended. Supraglottic devices (laryngeal mask, laryngeal tube) are highly efficient alternatives in the event of intubation failure. Prior to intubation, a ratio of two ventilations to every 30 chest compressions is recommended. After intubation, ventilations should be continuous at a rate of 8 to 10 per minute.^(20,23)^ Ventilation must be sufficient to produce visible chest rise, but hyperventilation should be avoided, as it can increase intrathoracic pressure, hindering venous return to the heart and impairing cardiac output during resuscitation.^(21)^

When adequate oxygenation and hemodynamic support are not achieved through conventional ventilation and cardiovascular support strategies, the use of venoarterial extracorporeal membrane oxygenation (ECMO), where available, has been reported. However, in cases of AFE complicated by DIC, the risk of bleeding during ECMO is significant, and systemic anticoagulation must be carefully weighed.^(24)^

### How should hemorrhage and coagulopathy be managed in amniotic fluid embolism?

Low levels of fibrinogen and factor V, associated with high D-dimer concentrations, are the classic alterations of hyperfibrinolysis, which is predominantly present in AFE. Conversely, a normal platelet count and slightly reduced antithrombin levels rule out massive activation of the coagulation system. In AFE, immediate and rapid massive transfusion is essential for patients with severe hemorrhage and/or DIC. Therefore, the institutional massive transfusion protocol must be activated immediately. Crystalloids (lactated Ringer’s and normal saline) should be infused until blood components become available. The recommended maximum crystalloid infusion is 1,500 mL, with the aim of preventing the worsening of coagulopathy due to hemodilution.^(25,26)^

Hemorrhagic etiology in AFE (the “T for Thrombin” component) requires blood transfusion as an effective and definitive treatment. Infusion of uterotonics, non-surgical invasive interventions (intrauterine balloons and intrauterine suction systems), and surgical invasive procedures (vascular ligation, uterine compression sutures, and hysterectomy) may also be used as adjuncts to control hemorrhage, but they are not considered definitive treatments.^(25,26)^

Hematologic values (hemoglobin and hematocrit) are not useful indicators of the acute blood loss characteristic of AFE. Vital signs must be monitored, and the shock index (heart rate/systolic blood pressure) should be used as a parameter to estimate blood loss, classify the degree of shock, monitor hemodynamics, and plan transfusion therapy. If hemorrhage is not controlled 30 minutes after the administration of tranexamic acid, which is part of initial management, a second dose (1 g intravenously over 10 minutes) should be infused.^(25,26)^

Massive transfusion protocols in resuscitation guidelines define the classic balanced ratio as 1:1:1 for packed red blood cells (PRBCs), fresh frozen plasma (FFP), platelets (PLT), with cryoprecipitate (CRYO) dosed separately alongside them. The most recommended protocol suggests four rounds (Chart 2).^(27)^

**Chart 2:** Ratios of blood components and tranexamic acid in massive transfusion rounds

	Packed red blood cells	Fresh frozen plasma	Platelets	Cryoprecipitate
Round 1	6 U	6 U	6 U	10 U
Round 2	6 U	6 U	6 U	10 U
Round 3	Tranexamic acid, 1 g intravenously, over 10 minutes			
Round 4	6 U	6 U	6 U	-

Source: Adapted from Pacheco et al. (2016).^(^^27^^)^

Therapeutic targets are hemoglobin > 8 g/dL, fibrinogen between 150 and 200 mg/dL, platelets > 50,000/mm^3^, and INR ≤ 1.5. In DIC, continuous platelet consumption correlates with a smaller increase in platelet counts. Patients with persistent platelet counts < 50,000/µL require transfusion optimization. Similarly, those with prolongation of prothrombin time or activated partial thromboplastin time require fresh frozen plasma to correct the INR. Fibrinogen levels remaining < 200 mg/dL indicate a need for fresh frozen plasma and cryoprecipitate transfusion. Each unit of cryoprecipitate raises the fibrinogen level by aproximately 10 mg/dL. However, since cryoprecipitate requires thawing, there is often a delay in its availability. Therefore, in patients maintaining fibrinogen levels < 50 mg/dL, fresh frozen plasma, if available in the massive transfusion cooler, should be administered immediately while awaiting the preparation of cryoprecipitate.^(25)^ When available, lyophilized fibrinogen concentrate is an alternative for early replacement.^(26,28)^

The use of fibrinogen and higher doses of tranexamic acid has also been proposed for treating AFE-associated coagulopathy. Fibrinogen (2 g) should be infused if hemorrhage persists after the initial round of PRBCs, FFP, and tranexamic acid, serving as a substitute for CRYO. Despite the higher cost, fibrinogen is less frequently associated with transfusion reactions. Continuous infusion (maintenance regimen) of tranexamic acid is feasible in cases of persistent hemorrhage requiring massive transfusion, as an early rise in D-dimer levels is observed in these situations. Thus, a simplified transfusion protocol, incorporating fibrinogen infusion and a tranexamic acid maintenance regimen, may also be adopted (Chart 3).^(25,29)^ Treating coagulopathy is justified even in the absence of active hemorrhage, particularly if the risk of bleeding is deemed high or if surgical procedures (e.g., cesarean section) are still required.

**Chart 3:** Ratios of blood components and tranexamic acid in simplified massive transfusion cycles for amniotic fluid embolism.

	Packed red blood cells	Fresh frozen plasma	Platelets	Fibrinogen	Tranexamic acid*
Round 1	2 U	2-3 U	0	0	1 g
Round 2	2 U	2-3 U	0	2 g	0
Round 3	2 U	U	2 U	1-2 g	1 g

* Alternative: 1 g, followed by a continuous infusion of 10–20 mg/kg body weight/hour, in case of persistent bleeding.

Source: Adapted from Schroeder et al. (2020).^(^^29^^)^

In the absence of thrombosis, prophylactic anticoagulation is not indicated for DIC. Recombinant human factor VII may be used in patients with severe coagulopathy and bleeding associated with childbirth. However, due to its potential association with thrombotic and thromboembolic events (stroke and pulmonary embolism), its use in AFE should be reserved as a last resort for hemorrhage refractory to invasive procedures.^(30)^

### When and how should fetal delivery be performed in amniotic fluid embolism?

Immediate delivery may be necessary in cases of AFE occurring after 22 to 23 weeks of gestation. This decision must be individualized, but the most common factors requiring urgent delivery are non-reassuring fetal status and rapid, progressive deterioration of the mother’s clinical condition. Fetal hypoxia is typically identified via fetal heart rate auscultation or Category III (abnormal/pre-terminal) cardiotocography tracings.^(31)^

In cases of maternal deterioration, particularly CPA, resuscitative hysterotomy (perimortem cesarean section) increases the chances of successful maternal resuscitation. Under these conditions, the procedure should be initiated four minutes after the onset of CPA if spontaneous circulation has not been restored, and fetal delivery should be completed within five minutes. In cases involving non-shockable rhythms, or when the duration of CPA is unknown or unwitnessed, surgery should be performed as soon as possible. In cases of CPA with shockable rhythms, the procedure should be initiated immediately following unsuccessful defibrillation.^(31)^ A scalpel and manual technique are the primary tools used for the hysterotomy. The abdominal incision may be a midline vertical incision or a low transverse incision (extended Joel-Cohen). In addition to speed, the midline vertical incision provides adequate uterine exposure, results in less bleeding, and offers access to the diaphragm, which may be useful for subsequent resuscitation interventions (such as direct cardiac massage). The hysterotomy should be a classic corporal incision (vertical, 5–7 cm starting from the uterine fundus). Only the fetus is removed and taken for drying, warming, and neonatal resuscitation. The uterine cavity must be immediately packed with surgical sponges, without removing the placenta, amniotic membranes, or umbilical cord. Left uterine displacement is discontinued, and CPR proceeds with the patient in the supine position. Once maternal cardiovascular stability is achieved, placental removal, hysterorrhaphy, and laparorrhaphy should be performed. The technical details of resuscitative hysterotomy that lack expert consensus and scientific evidence primarily concern aponeurosis suturing and placental removal followed by hysterorrhaphy. Aponeurosis suturing is intended to prevent abdominal extrusion of bowel loops during ongoing resuscitation maneuvers. Conversely, placental removal followed by hysterorrhaphy aims to reduce subsequent blood loss associated with the restoration of hemodynamic stability.^(32)^

For cesarean deliveries not associated with CPA, standard surgical techniques should be used, prioritizing strategies that reduce operative time and intraoperative bleeding. Aiming to prevent potential difficulties during fetal extraction, the transverse skin incision should be at least 15 cm long. Laparotomy should be performed using blunt dissection techniques (Joel-Cohen and Misgav-Ladach). Dissection of the bladder flap should be avoided. A high transverse segmental hysterotomy should be performed, with blunt cephalocaudal expansion carried out 5 cm above the vesicouterine reflection. Multi-layer hysterorrhaphy is recommended. Closure of the parietal peritoneum, rectus abdominis muscle, and subcutaneous layers less than 2 cm thick is unnecessary. Systematic suturing of the aponeurosis is required to prevent dehiscence and evisceration. Due to the coagulopathy associated with AFE, peritoneal drainage via a closed-suction drain is recommended.^(33)^

Broad-spectrum antibiotic prophylaxis and prophylactic oxytocin (continuous intravenous infusion, 20 mU/minute) are routinely recommended. Intravenous bolus injection of oxytocin is contraindicated due to the risk of hypotension, cardiac arrhythmias, cardiovascular collapse, and death.^(32,33)^

In cases of AFE occurring during the second stage of labor with the fetal head engaged, operative vaginal delivery using forceps or a vacuum extractor is appropriate.^(31)^

### What are the main differential diagnoses for amniotic fluid embolism?

Amniotic fluid embolism is typically a diagnosis of exclusion. Therefore, etiologies that also present with cardiopulmonary compromise and/or hemorrhage are considered differential diagnoses. Key examples include complications of neuraxial anesthesia; hemorrhage due to uterine atony; retained products of conception, and/or birth canal trauma; placental abruption; pulmonary thromboembolism; other conditions causing acute respiratory failure; air embolism; acute myocardial infarction; and anaphylactic, cardiogenic, or septic shock.^(2,9)^

Uterine atony, retained products of conception, and/or birth canal trauma are the primary hemorrhagic conditions to be differentiated from AFE. The key factor distinguishing AFE from these other causes of postpartum hemorrhage is the severity of the associated coagulopathy and the possibility of its onset during the antepartum period. Furthermore, uterine atony, genital tract lacerations, and/or retained placenta are more readily diagnosed via obstetric physical examination. Importantly, in cases of placental abruption, DIC may also manifest antepartum and is often disproportionate to the volume of vaginal blood loss. Placental abruption can be distinguished from AFE by the presence of abdominal pain and uterine hypertonia, as well as the absence of CPA or sudden hypotension.^(2,9)^

Pulmonary thromboembolism, anesthetic complications, acute myocardial infarction, and septic shock can also mimic AFE. The abrupt onset of symptoms, the occurrence of manifestations during labor or within 30 minutes of placental delivery, and the absence of fever are clinical criteria that can help differentiate AFE from these other etiologies.^(2,9)^

All other etiologies presenting with CPA, acute cardiopulmonary compromise, and hypoxemia during labor must be considered in the differential diagnosis of AFE. Key examples include acute pulmonary embolism, anaphylactic shock, septic shock, cardiogenic shock, anesthetic shock, air embolism, other causes of DIC (acute fatty liver of pregnancy and placental abruption), conditions leading to acute respiratory failure (pneumonia, aspiration, and trauma), and rare causes such as aortic dissection and tension pneumothorax. Chart 4 outlines the main clinical features and diagnostic criteria to be used in the differential diagnosis of AFE.^(2,9)^

**Chart 4:** Differential diagnosis of amniotic fluid embolism

Differential diagnosis	Clinical and ancillary findings	Diagnostic criteria
Acute pulmonary embolism	Sudden dyspnea, pleuritic chest pain, hemoptysis, history of or current venous thromboembolism. Unremarkable chest X-ray.	Echocardiography. Pulmonary CT angiography. Ventilation/perfusion scintigraphy: may be diagnostic in pregnant women but is not ideal in the setting of life-threatening anaphylactic shock. D-dimer measurement is not useful, as levels are elevated in late pregnancy and other acute intercurrent illnesses.
Anaphylactic shock	Sudden hypotension, evidence of allergy (labial and oropharyngeal edema, inspiratory stridor, and urticaria), exposure to a known inducing agent. DIC is unlikely.	Clinical diagnosis. Confirmation via elevated serum or plasma total tryptase or plasma histamine concentrations.
Anesthetic shock	History of local, spinal, or general anesthesia. DIC is rare.	Clinical diagnosis. Resolution of shock after discontinuation of anesthetic agents.
Cardiogenic shock	Chest pain, pre-existing cardiomyopathy, risk factors for ischemic heart disease or arrhythmia. Echocardiogram: regional wall motion abnormality, pericardial effusion, or valvular rupture. DIC is uncommon.	Echocardiography and electrocardiogram showing specific findings.
Hemorrhagic shock	Heavy vaginal bleeding. Specific signs and symptoms of placenta previa (painless bleeding), placental abruption (uterine hypertonia, pain), uterine atony, uterine rupture (intense pain followed by relief and shock; fetal presentation inaccessible), and genital tract laceration. Progression to DIC.	Clinical diagnosis. Placenta previa: ultrasound shows the placenta covering the internal cervical os. Placental abruption: bleeding precedes delivery and may persist postpartum. Uterine rupture: bleeding at the time of rupture, before or immediately after delivery.
Septic shock	Suspected source of infection, more insidious onset, fever, leukocytosis with left shift, hypovolemia.	Clinical diagnosis. Confirmation: cultures and response to antibiotics. Serum lactate measurement is unhelpful, as levels also rise in other forms of shock.
Air embolism	History of instrumentation (cesarean section, central venous catheter insertion). Visualization of air on imaging studies. DIC and hemorrhage are uncommon.	Clinical diagnosis. Demonstration of air in the intravascular space or organs.
DIC from other causes	Clear history of other causes: trauma, transfusion of blood products, placental abruption, preeclampsia, sepsis, intrauterine fetal demise, and acute fatty liver of pregnancy.	Clinical and laboratory diagnosis.
Hypoxemic respiratory failure	Respiratory distress syndrome with insidious onset, associated with bilateral pulmonary infiltrates. Precipitating factors (trauma, pneumonia). Evidence of aspiration (Mendelson’s syndrome). Delayed radiographic changes.	Clinical diagnosis. Identification of underlying etiologies.
Seizures	Eclampsia. Anesthetic medications.	Clinical diagnosis. Electrocardiogram with specific findings.
Rare causes (aortic dissection, cholesterol embolism, tension pneumothorax)	Aortic dissection: back or chest pain. Cholesterol embolism: known atherosclerosis or vascular manipulation. Pneumothorax: pleuritic chest pain, tracheal deviation, absence of breath sounds, history of cystic lung disease.	Echocardiography: aortic dissection. Lung ultrasound: tension pneumothorax. Cholesterol embolism: clinical diagnosis; vascular imaging.

DIC: disseminated intravascular coagulation.

Source: Adapted from Baldisseri and Clarck (2021).^(^^34^^)^

The severity of AFE, the wide range of differential diagnoses, and the challenges in confirming the diagnosis necessitate an extensive diagnostic workup. In addition to initial management, including CPR, control of hemorrhage and coagulopathy, and fetal delivery, the following diagnostic tests are recommended: blood typing and indirect Coombs test (antibody screen), complete blood count, coagulation profile, fibrinogen, renal function tests (urea and creatinine), electrolyte panel, serum calcium, magnesium, and phosphate levels, liver function tests, troponin-I, brain natriuretic peptide (BNP), arterial blood gas analysis, chest X-ray, electrocardiography, and ultrasonography. Ultrasonography, often performed at the bedside, should include assessment of the lower limbs, lungs, abdomen, and pelvis, as well as transthoracic echocardiography. Transesophageal echocardiography may also be useful, optimizing the detection of right ventricular failure and pulmonary hypertension.^(2,9,35)^

### How should patients with amniotic fluid embolism be managed following initial treatment

Following CPR, hemorrhage control, reversal of coagulopathy, and delivery, ongoing care for AFE patients should take place in an intensive care unit. Hemodynamically stable patients should receive supportive care focused on maintaining a secure airway, hemodynamic stability, and adequate oxygenation, as well as monitoring for and preventing hemorrhage and, if still necessary, investigating other etiologies.^(2,9,20)^

Stable patients without respiratory distress syndrome should undergo mechanical ventilation with tidal volumes of 6 to 8 mL/kg of predicted body weight. In the presence of respiratory distress, low tidal volume ventilation (4 to 6 mL/kg) is recommended. The use of positive end-expiratory pressure (PEEP) is not contraindicated. The ideal fraction of inspired oxygen (FiO_2_) is unknown, but peripheral oxygen saturation should be maintained above 96%. When available, ECMO oxygenation, intra-aortic balloon pumps, and cardiopulmonary bypass have been successfully used in cases progressing to respiratory failure or pulmonary hypertension.^(36,37)^

Clinical and hemodynamic monitoring of resuscitation measures is recommended. Once the patient is hemodynamically stable and volume replacement is complete, fluid infusion should be reduced or discontinued. Excessive fluid infusion must be avoided, as AFE can be associated with pulmonary edema. The use of vasopressors in unstable patients should be individualized; norepinephrine with or without dobutamine is the preferred option.^(20)^

Disseminated intravascular coagulation with persistent bleeding requires blood product transfusion, even in hemodynamically stable patients. In patients with DIC and controlled bleeding, the indication for transfusion should be individualized, taking into account the estimated risk of future bleeding.^(25,27)^

Obtaining central vascular access (venous and arterial) is recommended to allow for hemodynamic monitoring and frequent blood sampling for laboratory tests. Other intensive support measures include endotracheal tube care, measures to prevent ventilator-associated pneumonia, deep vein thrombosis and gastrointestinal prophylaxis, nutritional support, and glycemic control.^(9,18)^

Patients who remain unstable require intermittent laboratory assessments every four to six hours to monitor the therapeutic response. Key tests include a complete blood count (including platelets), coagulation profile, fibrinogen, liver and renal function tests, serum electrolytes, calcium, magnesium, phosphate, D-dimer levels, and arterial blood gas analysis.^(9,18)^

### What is the ideal sequence of interventions for managing amniotic fluid embolism?

Since AFE is an acute, highly lethal event requiring CPR, management of hemorrhage and coagulopathy, fetal delivery, and investigation of other serious differential diagnoses, flowcharts are useful for guiding the logical sequence of steps and decisions involved in its management. Flowchart 1 presents a sequence of suggested actions for the immediate care of pregnant women with suspected AFE.^(1)^

**Flowchart 1:**
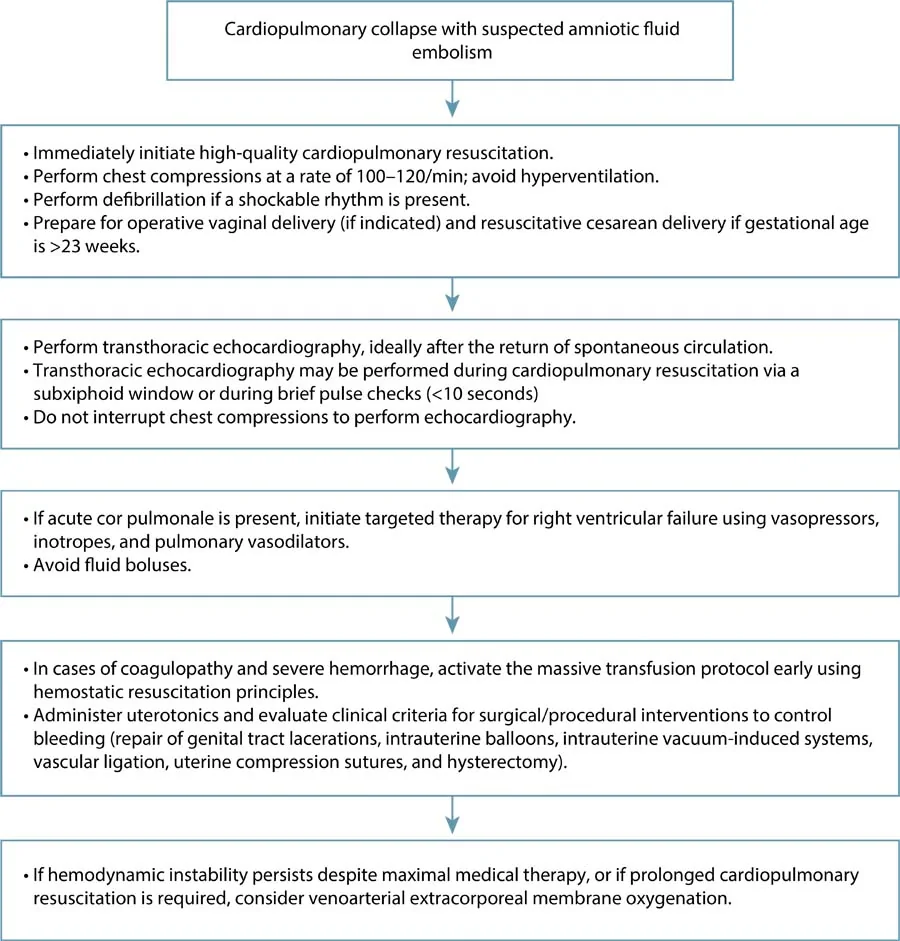
Sequence of actions in the immediate management of amniotic fluid embolism

Source: Adapted from Pacheco et al. (2020).^(^^1^^)^

## Final considerations

Despite its low incidence, AFE remains a challenging obstetric emergency with a high maternal mortality. The nonspecific nature of its diagnosis and the requirement for access to critical care are the primary management challenges, often leading to care delays and high fatality rates.^(1,2)^

Since AFE typically manifests during or immediately after delivery, presenting with CPA or sudden arterial hypotension associated with acute respiratory failure and DIC-induced hemorrhage, patient survival depends directly on the ability of healthcare facilities and care teams to rapidly provide BLS and ACLS alongside specialized obstetric interventions.^(1,2)^

To reduce AFE mortality, healthcare facilities must improve resource availability and team training for critically ill pregnant women. However, because AFE is so rare, maintaining team readiness and clinical expertise is inherently difficult. Therefore, the implementation of clinical guidelines, flowcharts, and multidisciplinary simulation training, is strongly recommended to structure care delivery and qualify care teams.^(1,2)^
